# An Investigation of the Antigastric Cancer Effect in Tumor Microenvironment of *Radix Rhei Et Rhizome*: A Network Pharmacology Study

**DOI:** 10.1155/2021/9913952

**Published:** 2021-06-24

**Authors:** Xinmiao Wang, Guanghui Zhu, Haoyu Yang, Ruike Gao, Zhe Wu, Ying Zhang, Xiaoyu Zhu, Xiaoxiao Zhang, Jie Li

**Affiliations:** ^1^Guang'anmen Hospital, China Academy of Chinese Medical Sciences, Beijing 100053, China; ^2^Beijing University of Traditional Chinese Medicine, Beijing 100029, China

## Abstract

**Background:**

Tumor microenvironment (TME) takes a vital effect on the occurrence and development of cancer. *Radix Rhei Et Rhizome* (RRER, Da-Huang in pinyin), a classical Chinese herb, has been widely used in gastric cancer (GC) for many years in China. However, inadequate systematic studies have focused on the anti-GC effect of RRER in TME. This study intended to uncover the mechanism of it by network pharmacology.

**Methods:**

We collected compounds and targets of RRER from traditional Chinese medicine system pharmacology database and analysis platform (TCMSP) and SwissTargetPrediction. GC targets were obtained from GeneCards. Protein-protein interaction (PPI) network and RRER-GC-target network were built by STRING and Cytoscape 3.2.1. Furthermore, Gene Ontology (GO) and Kyoto Encyclopedia of Genes and Genomes (KEGG) analysis were performed using Database for Annotation, Visualization, and Integrated Discovery (DAVID).

**Results:**

We obtained 92 compounds of RRER. A total of 10 key compounds and 20 key targets were selected by “RRER-GC-target network” topological analysis. GO analysis showed that the biological process mainly involved in response to the tumor necrosis factor, positive regulation of fibroblast proliferation, and DNA damage response, signal transduction by p53 class mediator resulting in cell cycle arrest. Molecular functions included cyclin-dependent protein serine/threonine kinase activity, RNA polymerase II transcription factor activity, ligand-activated sequence-specific DNA binding, and transmembrane receptor protein tyrosine kinase activity. Cellular components mainly were centrosome, cell surface, and membrane. KEGG pathway enrichment results mainly involved in the p53 signaling pathway, estrogen signaling pathway, and regulation of lipolysis in adipocytes.

**Conclusion:**

This study explored the anti-GC mechanism of RRER from the perspective of TME based on network pharmacology, which contributed to the development and application of RRER.

## 1. Introduction

Gastric cancer (GC) remains the world's second most common cause of cancer-related death [1]. There are nearly 1 million new GC cases in the world each year [2]. Once suffering from this disease, it will be followed by the patient's physical injury, mental pressure, and health expenditure, which bring a heavy burden to both society and individuals. At present, the main treatments of GC include surgery, chemotherapy, chemoradiotherapy, and targeted and immune therapies, among which surgery is the only chance to cure, but recurrence is common.

In recent years, *Radix Rhei Et Rhizome* (RRER, Da-huang in pinyin), a classic Chinese herb, has been widely used in GC. For example, RRER-Zhechong pill and RRER-Renshen decoction with chemotherapy are used in advanced GC [3, 4], and RRER-Huanglian-Xiexin decoction is applied to cure GC precancerous lesions [5]. Moreover, a single RRER has been used in GC complicated with hemorrhage [6]. Besides, the methanol extract of RRER has the anti-GC effect by mediating cell death with an intrinsic apoptotic pathway [7]. Its active compound, aloe-emodin, has growth inhibitory effects in GC cells with an increase in S phase and alkaline phosphatase activity repression [8].

Notably, GC is not a simple mass of tumor cells. It is located in a very complex system named tumor microenvironment (TME), which includes the tumor vasculature, composition of the extracellular matrix, and stroma cells. A compelling body of evidence has demonstrated that TME takes a vital effect on the occurrence and development of GC [9–11]. However, due to the diversity of traditional Chinese medicine (TCM) compounds, inadequate systematic studies have focused on the anti-GC effect of RRER in TME.

Network pharmacology, first proposed by Hopkins [12], is a new discipline covering systems biology and network analysis. It emphasizes that drugs are mainly obtained through multitarget interaction to take the synergistic effect. This viewpoint is coincident with the TCM's characteristic of multicompounds and multitargets. At present, there have been many studies [13–15] providing useful examples for exploring the TCM mechanism by means of network pharmacology at home and abroad. Therefore, this study intended to use network pharmacology to uncover the underlying anti-GC mechanism of RRER in TME.

## 2. Materials and Methods

### 2.1. Research Tools

The traditional Chinese medicine system pharmacology database and analysis platform [16] (TCMSP, http://lsp.nwu.edu.cn/tcmsp.php, version: 2.3) and SwissTargetPrediction [17] (http://www.swisstargetprediction.ch/, updated in 2019) were used to collect RRER compounds and targets. GeneCards [18] (https://www.genecards.org/, version: 5.0) was applied to get GC targets. UniProt [19] (https://www.uniprot.org/) was selected to supplement the targets' UniProt ID. STRING [20] (http://string-db.org, version 11.0) was used to establish the protein-protein interaction (PPI) network. Cytoscape 3.2.1 [21] (http://www.cytoscape.org/) was applied to construct the RRER-GC-target network and do network topology analysis. Database for Annotation, Visualization, and Integrated Discovery [22] (DAVID, http://david.ncifcrf.Gov, version: 6.8) was used for Gene Ontology (GO) and Kyoto Encyclopedia of Genes and Genomes (KEGG) analysis. OmicShare (https://www.Omicshare.com/) was selected to visualize the GO and KEGG results.

### 2.2. Collection and Screening of the RRER Active Compounds

TCMSP is a systems pharmacology platform of Chinese herbal medicines that collects chemicals, targets, and pharmacokinetic properties of natural compounds involving oral bioavailability (OB), drug-likeness (DL), intestinal epithelial permeability, blood-brain barrier, and aqueous solubility. Among them, OB [23] is the rate and extent to which the active ingredient is absorbed from a drug product and becomes available at the site of action, and DL [24] means that drugs and drug candidates tend to have similar physicochemical properties. Both of them are important references for evaluating whether a compound has the potential to become a drug [25, 26]. In this study, we used TCMSP to search the RRER compounds and canonical SMILES and then took “OB ≥ 30% and DL ≥ 0.18” as the screening standard to filter the active compounds.

### 2.3. Prediction of RRER and GC Targets

First, to predict RRER's targets, we uploaded canonical SMILES to the SwissTargetPrediction database, saved targets' full name, abbreviation, and UniProt ID and then eliminated the repeated targets. When the results of the predicted targets were 0, we used TCMSP to supplement them. Second, we used “gastric cancer” as the keyword to search disease targets in the GeneCards database. After removing targets with relevance score less than 20 and supplementing targets' UniProt ID in the UniProt database, we finally obtained GC-related targets' full names, abbreviations, and UniProt IDs.

### 2.4. Network Construction and Analysis

By mapping RRER targets and GC targets, we obtained RRER and GC common targets and then the constructed PPI network (combined score ≥0.9) through the STRING database. Simultaneously, the RRER-GC-common target network was built by Cytoscape 3.2.1. After that, network topological analysis was carried out to find the pivotal nodes in the network.

### 2.5. GO and KEGG Analysis

Go is a gene function classification that describes the properties of genes and gene products. There are 3 ontologies in GO, which include the molecular function, cellular component, and biological process. In this study, we used DAVID to carry out GO and KEGG analysis by importing the key targets into “Functional Annotation.” And then, the OmicShare tool was used to visualize the analysis results.

## 3. Results

### 3.1. Collection and Screening the RRER Active Compounds

We obtained 92 active compounds of RRER after searching in the TCMSP database, among which 16 active compounds met the screening criteria of “OB ≥ 30%, DL ≥ 0.18” (Table 1).

### 3.2. Prediction of RRER and GC Targets

We obtained 583 targets of RRER by SwissTargetPrediction. Because results of the 2 active compounds (aloe-emodin and (-)-catechin) were 0 in SwissTargetPrediction, we used TCMSP to supplement them and then added 34 targets. Finally, we totally collected 617 RRER's targets (Table 1). A total of 11 842 GC targets were obtained in GeneCards. Furthermore, with the filtering criteria of “relevance score ≥20,” a total of 448 GC-related targets were collected (Supplementary Table S1).

### 3.3. Construction and Analysis of Networks

**Figure 1:**
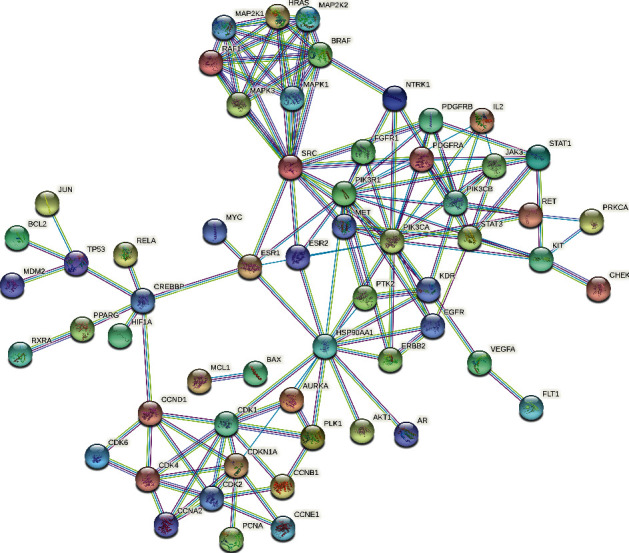
PPI network (combined score ≥0.9) of common targets. Nodes represent proteins. Edges represent interactions between protein and protein.

**Figure 2:**
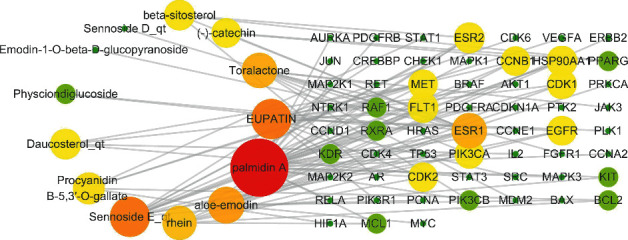
RRER-GC-common target network. The nodes' color and size are determined by degree. The larger and the redder the node, the higher the degree it is.

After mapping RRER targets and GC targets, a total of 99 common targets were obtained. To better understand the interactions between common targets, a PPI network (combined score ≥0.9) was built by STRING (Figure 1). Based on the targets (combined score ≥0.9) obtained in Figure 1, a RRER-GC-common target network was constructed by Cytoscape 3.2.1 (Figure 2). The network topology analysis showed that top 10 degree of RRER's compounds included palmidin A, eupatin, sennoside E_qt, aloe-emodin, toralactone, rhein, procyanidin B-5,3′-O-gallate, daucosterol_qt, beta-sitosterol, and (-)-catechin (Table 2). Top 20 degree of common targets were ESR1, ESR2, HSP90AA1, FLT1, CCNB1, CDK1, EGFR, MET, CDK2, PIK3CA, KDR, BCL2, MCL1, RAF1, RXRA, KIT, PIK3CB, PPARG, PIK3R1, and PLK1 (Table 3).

### 3.4. GO and KEGG Analysis

**Figure 3:**
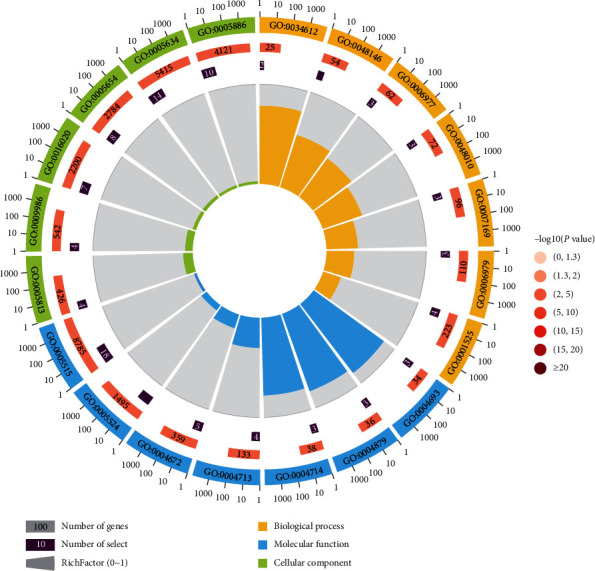
GO analysis of key targets. There are four circles in the figure. From outside to inside, the first circle is the classification of enrichment. Different colors represent different classifications. The second circle shows the number of background genes and *P* value. The more genes, the longer the bars; the smaller the *P* value, the redder the color. The third circle is the total number of prospective genes. The forth circle represents the RichFactor, which indicates ratio of genes in the current study versus the total genes in the term. GO:0005634, nucleus; GO:0005813, centrosome; GO:0005886, plasma membrane; GO:0009986, cell surface; GO:0005654, nucleoplasm; GO:0016020, membrane; GO:0004879, RNA polymerase II transcription factor activity, ligand-activated sequence-specific DNA binding; GO:0004714, transmembrane receptor protein tyrosine kinase activity; GO:0005524, ATP binding; GO:0004713, protein tyrosine kinase activity; GO:0004672, protein kinase activity; GO:0004693, cyclin-dependent protein serine/threonine kinase activity; GO:0005515, protein binding; GO:0048146, positive regulation of fibroblast proliferation; GO:0001525, angiogenesis; GO:0006977, DNA damage response, signal transduction by p53 class mediator resulting in cell cycle arrest; GO:0048010, vascular endothelial growth factor receptor signaling pathway; GO:0007169, transmembrane receptor protein tyrosine kinase signaling pathway; GO:0006979, response to oxidative stress; GO:0034612, response to the tumor necrosis factor.

GO analysis showed that the biological process mainly involved in response to the tumor necrosis factor, positive regulation of fibroblast proliferation, and DNA damage response, signal transduction by p53 class mediator resulting in cell cycle arrest. Molecular functions included the cyclin-dependent protein serine/threonine kinase activity, RNA polymerase II transcription factor activity, ligand-activated sequence-specific DNA binding, and transmembrane receptor protein tyrosine kinase activity. Cellular components mainly were centrosome, cell surface, and membrane (Figure 3).

**Figure 4:**
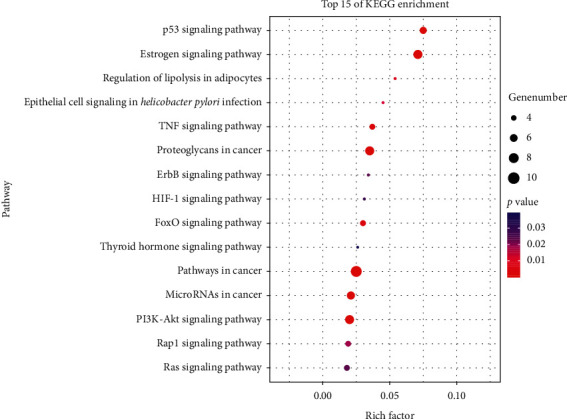
Top 15 KEGG pathway enrichments. Node color is displayed in a gradient from red to green in descending order of the *P* value. The size of the nodes is arranged in ascending order according to the number of genes. RichFactor is the ratio of genes in the current study versus the total genes in the term.

There were 36 KEGG pathways obtained by DAVID, among which 32 pathways' *P* values were less than 0.05 (Supplementary Table S2). As shown in Figure 4, pathways with high rich factors include the p53 signaling pathway, estrogen signaling pathway, regulation of lipolysis in adipocytes, epithelial cell signaling in *Helicobacter pylori* infection, TNF signaling pathway, proteoglycans in cancer, HIF-1 signaling pathway, FoxO signaling pathway, thyroid hormone signaling pathway, MicroRNAs in cancer, and PI3K-Akt signaling pathway.

## 4. Discussion

RRER has been widely applied in gastrointestinal diseases for many years. In TCM's theory, it is a bitter, cold dry herb used to “clear heat” from the liver, stomach, and blood [27]. Based on network pharmacology, this study was to uncover the targets and molecular mechanisms exerted by RRER in the TME of GC. In “RRER-GC-common target network,” one active compound could act on several targets, and the same target could be linked to different active compounds, indicating the multitarget and synergistic strategy of RRER. In the top 10 degree of compounds, three of them have been verified to have anti-GC effects. For example, aloe-emodin could arrest the cell cycle of MKN45 human GC cells in G0/G1 phase or G0/G1 and G2/M phases [28]. Rhein could induce apoptosis of human GC SGC-7901 cells [29]. Beta-sitosterol has an antitumor effect in AGS human gastric adenocarcinoma cells and xenograft mouse models [30]. These results are consistent with our predictions, suggesting that high-degree compounds might play an important role in the treatment of GC.

The occurrence and development of the tumor is not only related to its own malignant proliferation but also closely related to TME [31]. TME is a highly dynamic and heterogeneous composition of immune cells, fibroblasts, precursor cells, endothelial cells, signaling molecules, and extracellular matrix components [32]. COX-2 (also known as PTGS2), released by cancer-associated fibroblasts (CAFs) [33] and macrophage type 2 cells [34], is one of the key markers predicting worse cancer prognosis and stimulates cancer via multiple ways in the TME [35]. Study has shown that COX-2 inhibitors have the potential to decrease the risk of tumorigenesis [36]. RRER's main compounds, aloe-emodin and (-)-catechin, can suppress the level of PTGS2 [37, 38]. Interestingly, rhein can inhibit EGFR [39], the upstream modulator of COX-2 in cancer cells [40]. Accordingly, we speculate that rhein might play an important role in TME by targeting EGFR and its downstream target COX-2.

The result of GO analysis is also consistent with our prediction. For example, the biological process of RRER includes fibroblast proliferation, which is verified to play an important role in TME [41]. KEGG pathway analysis shows that RRER involves various pathways closely related to TME. (1) Epithelial cell signaling in *Helicobacter pylori* infection pathway: *H. pylori* exposure results in a chronic inflammation microenvironment which is strongly linked to GC [31]. Study has shown that the extract of RRER has the antibacterial activity against *H. pylori* both in vitro and in vivo. And the in vivo studies prove that RRER is highly efficient in terms of dosage, tolerability, and curing active *H. pylori* infection [27]. (2) PI3K-Akt signaling pathway: activation of the PI3K-Akt signaling pathway induces the process of EMT [42] and causes immune suppression and evasion in the TME [43]. It is reported that emodin can inhibit the PI3K-Akt signaling pathway and decrease tumor growth [44]. (3) HIF-1 signaling pathway: low oxygen tension (hypoxia) is an important component of TME as it alters the extracellular matrix, modulates the tumour immune response, and increases angiogenesis [45]. Emodin and rhein can decrease HIF-1*α* expression and attenuate cancer cachexia in athymic mice carrying cancer cells [46].

In conclusion, increasing evidence supports the reliability of the network pharmacology method, which may be an effective way to study the pharmacological mechanisms of Chinese herbs. This study explored the anti-GC mechanism of RRER from the perspective of TME based on network pharmacology, which might contribute to the development and application of RRER. However, some results of our study have not been fully confirmed at present. Future in vitro and in vivo studies will help to give more insight into unveiling the molecular mechanisms of RRER in TME.

## Figures and Tables

**Table 1:** Active compounds and targets of RRER.

No.	Active compounds	OB (%)	DL	Targets
1	Eupatin	50.8	0.41	XDH, CYP1B1, AKR1B1, PLG, OPRD1, MAPT, KDM4E, GPR35, AVPR2, TOP2A, CYP19A1, DRD4, GLO1, MPO, PIK3R1, DAPK1, PYGL, CA3, ABCC1, PLK1, CA6, PKN1, CSNK2A1, NEK2, CAMK2B, ALK, AKT1, NEK6, PLA2G1B, APEX1, NUAK1, AKR1C2, AKR1C1, AKR1C3, AKR1C4, AKR1A1, CA2, CA12, ALOX5, GSK3B, HSD17B2, ABCG2, CCNB1, CCNB3, CCNB2, CDK1, CDK6, ARG1, ADORA3, BACE1, APP, CA7, ADORA1, MMP3, MMP2, NOX4, EGFR, PIK3CG, MAOA, TYR, AHR, ESRRA, MET, FLT3, ADORA2A, KDR, IGF1R, INSR, SRC, PTK2, CA1, CA13, MMP13, CA4, MMP9, ALOX12, AURKB, ST6GAL1, CDK2, HSD17B1, CA9, PTPRS, MPG, SLC22A12, AXL, ABCB1, ODC1, PFKFB3, F2, CA14, CA5A, CD38, AKR1B10, TNKS2, TNKS, TOP1, MYLK, ALOX15, PIM1, CXCR1, PLA2G2A, ACHE, SYK
2	Mutatochrome	48.64	0.61	ALOX5
3	Physciondiglucoside	41.65	0.63	ESR1, TNNC1, TNNT2, TNNI3, EPHX2, SLC5A1, CA14, LGALS3, LGALS9, SLC5A2, CHIA, SLC29A1, ADORA2A, CYP19A1, ADORA3, MME, ECE1, LGALS4, LGALS8, SLC5A4, ACE, HRAS, ADORA2B, TYR
4	Procyanidin B-5,3′-O-gallate	31.99	0.32	MMP2, MAPT, DYRK1A, KCNH2, MAPK14, TERT, PGD, ST3GAL3, FUT7, BCL2, FUT4, STAT1, SQLE, BACE1, APP, MMP14, MET, ABCB1, DNMT1, MMP9, GABRA1, GABRB2, GABRG2, MMP12, PGF, VEGFA, HIF1A, CA2, CA1, CA9, ABCC1, ABCG2, PTGS1, CYP19A1, KLK1, KLK2, POLB, PLA2G2A, PLA2G5, PLA2G10, CYP1B1
5	Rhein	47.07	0.28	FTO, CYP19A1, ELANE, FNTA, FNTB, PTP4A3, CSNK2A1, ESR2, PIM1, CASP3, LDHA, LDHB, ERN1, ESR1, CDC25 B, BCL2, MCL1, AMPD3, ECE1, LIMK1, F2, SLC13A5, LCK, IGFBP3, GRK6, EGLN1, MME, CDK2, HNF4A, MAPK8, OGA, GPR35, ADA, ACLY, CASP6, CASP7, CASP8, CASP1, CASP2, NOX4, CAMKK2, ERBB2, SLC6A3, EGFR
6	Sennoside E_qt	50.69	0.61	FTO, ELANE, AKR1B1, CYP19A1, PIM1, CA2, CA1, TOP1, SELL, SELE, SELP, PTP4A3, CSNK2A1, BMP1, AMPD3, HSP90AB1, ACE, MME, PDE5A, MMP9, MMP1, MMP2, MMP8, ESR1, ESR2, HSP90AA1, KDM4C, PTGDR2, ECE1, PTGER1, PTGER2, IKBKB, PTGER3, AMPD2, PIK3CA, FNTA, FNTB, KDM3A, HCAR2, ITGB1, AGTR1, LTA4H, ITGB7, ITGA4, HTR2B, RAF1, SLC5A2, BRAF, EGLN1, TTL, MAPK8, PTGFR, MMP10, MMP12, TKT, FOLH1, RXRA, HNF4A, FLT1, KDM4A, MMP14, CXCR2, PYGL, PNP, CASP3, PTGER4, IGFBP3, MKNK2, EGFR, ITGAV, ITGB3, ADAMTS4, CASP6, CASP7, CASP8, CASP1, F7, CREBBP, FYN, OPRM1, IGFBP5, TTR, CHEK1, WEE1, KIT, CTSD, DYRK2, GSK3B, DPP4, GSK3A, AKR1B10, PDE4B, AMPD1, PDE4D, ROCK1, SCN9A, PTGIR, P2RX3, KCNH2, ACLY, CDK5, BCL2L2
7	Torachrysone-8-O-beta-D-(6′-oxayl)-glucoside	43.02	0.74	EPHX2, TYR, SRD5A1, TDP1, SLC5A2, PTPN1, SLC5A1, SLC5A4, CA14, ADORA2A, SLC29A1, HK2, HK1, AKR1B1, PYGL, ADORA3, EIF4H, PABPC1, PIM1, FUCA1, ADORA2B, NR4A1, IGFBP3
8	Toralactone	46.46	0.24	PTGS2, GUSB, SERPINE1, FADS1, CA1, CA12, CA9, IMPDH2, RET, QPCT, TYMS, PLAU, PDE5A, PTK2B, ABCB1, MTOR, PIK3CD, PIK3CB, HCK, PIK3CA, DYRK1B, JAK3, PLA2G7, ILK, TUBB1, TUBB3, RPS6KA3, AKR1B1, EGLN1, FLT1, DHFR, EPHB2, MDM2, MAOA, RAF1, FGFR1, CXCR2, MAOB, LNPEP
9	Emodin-1-O-beta-D-glucopyranoside	44.81	0.8	ESR1, TNNC1, TNNT2, TNNI3, EPHX2, SLC5A4, SLC5A2, SLC5A1, CA7, CA4, ELANE, SLC29A1, SLC28A3, ACHE, NQO2, PTPN1
10	Sennoside D_qt	61.06	0.61	TNNC1, TNNT2, TNNI3, ESR1
11	Daucosterol_qt	35.89	0.7	IL2, STAT3, APH1B, PSEN1, APH1A, NCSTN, PSENEN, PSEN2, PTAFR, MET, S1PR3, S1PR1, FLT1, RBP4, PPM1B, PPP1CC, PPP2CA, PPP2R5A, HSD11B2, S1PR5, S1PR4
12	Palmidin A	32.45	0.65	PTP4A3, PIM1, CSNK2A1, FTO, ESR1, ESR2, MAP3K14, MAP3K7, HSP90AA1, HDAC6, HDAC2, HDAC1, HMGCR, CXCR2, CXCR1, RXRA, TOP1, PARP1, PDK1, HNF4A, PDE5A, KIT, FLT3, KDR, MAP2K2, MAPKAPK5, TYK2, MAPK1, ALK, MMP9, MMP1, MMP2, MMP8, ACVRL1, ADORA3, MMP13, MMP3, ADAM17, CTSV, PLK4, CDK5, MKNK2, AXL, SORD, CDK2, CCND1, CDK4, SYK, GSK3B, GSK3A, MCL1, MMP7, MMP12, ELANE, ADCY1, MAP2K7, MAP2K1, RELA, FLT1, PDGFRB, FLT4, PDGFRA, MAPK3, PLG, APH1B, PSEN1, APH1A, NCSTN, PSENEN, PSEN2, PLAU, IRAK4, PRKCB, CCNB2, CCNB1, CDK1, CCNB3, CCNA2, CCNA1, RPS6KA3, AKR1B1, PDE4B, PIK3CD, PIK3CB, PIK3CG, MAP3K1, PIK3CA, IMPDH1, AURKA, P2RX7, CA2, OPRK1, CA1, CA12, CA9, JUN, P2RX3, PRKCD, NTRK1, GYS1, BACE1, LCK, CA4, WEE1, CCNE1, BCHE, CCNH, CDK7, CCNT1, CDK9, DYRK1A
13	Beta-sitosterol	36.91	0.75	AR, HMGCR, CYP51A1, NPC1L1, NR1H3, CYP19A1, CYP17A1, RORC, ESR1, ESR2, SREBF2, SHBG, SLC6A2, CYP2C19, RORA, PTPN1, BCHE, SERPINA6, SERPINA6, SLC6A4, CHRM2, VDR, ACHE, G6PD, NR1H2, GLRA1, CES2, PTGER1, PTGER2, HSD11B1, PTGES, CDC25 A, PPARA, PPARD, DHCR7, SQLE, PTPN6, NR1I3, FDFT1, SIGMAR1, NOS2, NR3C1, PPARG, CDC25 B, UGT2B7, HSD11B2, POLB
14	Aloe-emodin	83.38	0.24	PTGS1, PTGS2, HSP90AB1, HSP90AA1, PIK3CG, NCOA2, PKIA, AKR1B1, IGHG1, CDKN1A, EIF6, BAX, TNF, CASP3, TP53, FASN, PRKCA, PRKCE, CDK1, PCNA, MYC, IL1B, PRKCD, CCNB1
15	Gallic acid-3-O-(6′-O-galloyl)-glucoside	30.25	0.67	TDP1, SERPINE1, PTPN2, BACE1, ADORA1, AKR1B1, ASNS, AMY1A
16	(-)-Catechin	49.68	0.24	PTGS1, ESR1, PTGS2, HSP90AB1, HSP90AA1, DPEP1, NCOA2, FASN, PPARG, KLF7

**Table 2:** Network topology analysis of compounds (top 10 of degree).

No.	Effective compounds	Degree	Average shortest path length	Closeness centrality	Neighborhood connectivity	Radiality
1	Palmidin A	27	2.18309859	0.45806452	2.07407407	0.8028169
2	Eupatin	12	2.85915493	0.34975369	1.91666667	0.69014085
3	Sennoside E_qt	12	2.6056338	0.38378378	2.91666667	0.73239437
4	Aloe-emodin	9	3.16901408	0.31555556	1.77777778	0.63849765
5	Toralactone	8	3.42253521	0.29218107	8	0.57276995
6	Rhein	7	3.05633803	0.32718894	8	0.57276995
7	Procyanidin B-5,3′-O-gallate	5	3.95774648	0.25266904	4.66666667	0.61502347
8	Daucosterol_qt	4	3.1971831	0.31277533	4.5	0.57746479
9	Beta-sitosterol	4	3.47887324	0.28744939	1.875	0.59624413
10	(-)-Catechin	3	3.30985915	0.30212766	3.28571429	0.657277

**Table 3:** Network topology analysis of key targets (top 20 of degree).

No.	Targets	Degree	Average shortest path length	Closeness centrality	Neighborhood connectivity	Radiality
1	ESR1	8	2.57746479	0.38797814	7.125	0.7370892
2	ESR2	4	2.71830986	0.36787565	12.5	0.71361502
3	HSP90AA1	4	2.74647887	0.36410256	12.75	0.70892019
4	FLT1	4	2.63380282	0.37967914	12.75	0.72769953
5	CCNB1	3	2.6056338	0.38378378	16	0.73239437
6	CDK1	3	2.6056338	0.38378378	16	0.73239437
7	EGFR	3	3.02816901	0.33023256	10.33333333	0.66197183
8	MET	3	3.45070423	0.28979592	7	0.5915493
9	CDK2	3	2.71830986	0.36787565	15.33333333	0.71361502
10	PIK3CA	3	2.85915493	0.34975369	15.66666667	0.69014085
11	KDR	2	2.8028169	0.35678392	19.5	0.69953052
12	BCL2	2	3.81690141	0.26199262	6	0.53051643
13	MCL1	2	2.94366197	0.33971292	17	0.67605634
14	RAF1	2	3.42253521	0.29218107	10	0.59624413
15	RXRA	2	3	0.33333333	19.5	0.66666667
16	KIT	2	3	0.33333333	19.5	0.66666667
17	PIK3CB	2	3	0.33333333	17.5	0.66666667
18	PPARG	2	4.21126761	0.23745819	3.5	0.46478873
19	PIK3R1	1	3.84507042	0.26007326	12	0.5258216
20	PLK1	1	3.84507042	0.26007326	12	0.5258216

## Data Availability

The data used to support the results of this study are available from the corresponding author upon request.

## Abbreviations

CAFs: Cancer-associated fibroblasts
KEGG: Kyoto Encyclopedia of Genes and Genomes
DL: Drug-likeness
GC: Gastric cancer
GO: Gene Ontology
DAVID: Database for Annotation, Visualization, and Integrated Discovery
OB: Oral bioavailability
PPI: Protein-protein interaction
RRER: Radix Rhei Et Rhizome
TCM: Traditional Chinese medicine
TCMSP: Traditional Chinese medicine system pharmacology database and analysis platform
TME: Tumor microenvironment.

## Supplementary Materials

Supplementary Materials. Supplementary Table S1. GC-related targets. Supplementary Table S2. KEGG pathway analysis.
