# Supplementary material for: An Investigation of the Antigastric Cancer Effect in Tumor Microenvironment of Radix Rhei Et Rhizome: A Network Pharmacology Study
Source: Evid Based Complement Alternat Med. 2021 Jun 24;2021:9913952. doi: 10.1155/2021/9913952 (PMC8249119; doi:10.1155/2021/9913952)
Supplement: Supplementary Materials. — Supplementary Table S1. GC-related targets. Supplementary Table S2. KEGG pathway analysis. [file 9913952.f1.zip › 9913952.f1/Supplementary Table S1. GC-related targets.pdf]

Supplementary Table S1. GC-related targets

Gene Symbol	Description	uniprot ID	Category	Gifts	GC Id	Relevance score
CDH1	Cadherin 1	P12830	Protein Coding	50	GC16P068737	242.75
APC	APC Regulator Of WNT Signaling Pathway	P25054	Protein Coding	48	GC05P112707	166.36
BRCA2	BRCA2 DNA Repair Associated	P51587	Protein Coding	49	GC13P032315	152.9
MLH1	MutL Homolog 1	P40692	Protein Coding	48	GC03P036993	149.9
BRCA1	BRCA1 DNA Repair Associated	P38398	Protein Coding	50	GC17M043044	149.46
MSH2	MutS Homolog 2	P43246	Protein Coding	48	GC02P047402	148.84
TP53	Tumor Protein P53	P04637	Protein Coding	54	GC17M007661	146.04
ATM	ATM Serine/Threonine Kinase	Q13315	Protein Coding	54	GC11P108222	135.81
KRAS	KRAS Proto-Oncogene, GTPase	P01116	Protein Coding	51	GC12M025204	109.52
ERBB2	Erb-B2 Receptor Tyrosine Kinase 2	P04626	Protein Coding	54	GC17P039687	103.89
MSH6	MutS Homolog 6	P52701	Protein Coding	50	GC02P047695	100.15
PTEN	Phosphatase And Tensin Homolog	P60484	Protein Coding	52	GC10P087863	100.07
EGFR	Epidermal Growth Factor Receptor	P00533	Protein Coding	54	GC07P055019	99.34
CHEK2	Checkpoint Kinase 2	O96017	Protein Coding	53	GC22M028687	97.2
CDKN2A	Cyclin Dependent Kinase Inhibitor 2A	P42771	Protein Coding	51	GC09M021967	96.13
MET	MET Proto-Oncogene, Receptor Tyrosine Kinase	P08581	Protein Coding	54	GC07P116672	92.79
MUTYH	MutY DNA Glycosylase	Q9UIF7	Protein Coding	44	GC01M045329	90.16
STK11	Serine/Threonine Kinase 11	Q15831	Protein Coding	49	GC19P001177	88.53
PIK3CA	Phosphatidylinositol-4,5-Bisphosphate 3-Kinase Catalytic Subunit Alpha	P42336	Protein Coding	52	GC03P179148	83.21
CTNNB1	Catenin Beta 1	P35222	Protein Coding	53	GC03P041236	82.67
IL1B	Interleukin 1 Beta	P01584	Protein Coding	48	GC02M112829	81.57
SMAD4	SMAD Family Member 4	Q13485	Protein Coding	50	GC18P051028	81.39
MYC	MYC Proto-Oncogene, BHLH Transcription Factor	P01106	Protein Coding	51	GC08P127735	78.19
RB1	RB Transcriptional Corepressor 1	P06400	Protein Coding	49	GC13P048303	76.35
CCND1	Cyclin D1	P24385	Protein Coding	52	GC11P069641	76.01

PMS2	PMS1 Homolog 2, Mismatch Repair System Component	P54278	Protein Coding	48	GC07M005973	75.49
VEGFA	Vascular Endothelial Growth Factor A	P15692	Protein Coding	48	GC06P043770	75.38
CDKN1B	Cyclin Dependent Kinase Inhibitor 1B	P46527	Protein Coding	48	GC12P012716	75
AKT1	AKT Serine/Threonine Kinase 1	P31749	Protein Coding	54	GC14M104769	74.34
TGFBR2	Transforming Growth Factor Beta Receptor 2	P37173	Protein Coding	51	GC03P030623	73.69
HRAS	HRas Proto-Oncogene, GTPase	P01112	Protein Coding	52	GC11M000635	73.56
PALB2	Partner And Localizer Of BRCA2	Q86YC2	Protein Coding	43	GC16M023603	72.3
BRAF	B-Raf Proto-Oncogene, Serine/Threonine Kinase	P15056	Protein Coding	54	GC07M140719	71.51
TERT	Telomerase Reverse Transcriptase	O14746	Protein Coding	51	GC05M001253	71.39
KIT	KIT Proto-Oncogene, Receptor Tyrosine Kinase	P10721	Protein Coding	53	GC04P054657	70.91
TNF	Tumor Necrosis Factor	P01375	Protein Coding	51	GC06P047305	70.48
CDK4	Cyclin Dependent Kinase 4	P11802	Protein Coding	54	GC12M057743	70.44
NF1	Neurofibromin 1	P21359	Protein Coding	48	GC17P031094	69.46
CTNNA1	Catenin Alpha 1	P35221	Protein Coding	47	GC05P138613	68.05
EGF	Epidermal Growth Factor	P01133	Protein Coding	51	GC04P109912	67.87
CDKN1A	Cyclin Dependent Kinase Inhibitor 1A	P38936	Protein Coding	50	GC06P047460	67.16
IL6	Interleukin 6	P05231	Protein Coding	50	GC07P022765	66.09
BMPR1A	Bone Morphogenetic Protein Receptor Type 1A	P36894	Protein Coding	51	GC10P086756	65.9
AXIN2	Axin 2	Q9Y2T1	Protein Coding	48	GC17M065528	65.37
BAX	BCL2 Associated X, Apoptosis Regulator	Q07812	Protein Coding	48	GC19P048954	65.34
ESR1	Estrogen Receptor 1	P03372	Protein Coding	53	GC06P151656	64.88
STAT3	Signal Transducer And Activator Of Transcription 3	P40763	Protein Coding	52	GC17M042313	64.19
SMARCA4	SWI/SNF Related, Matrix Associated, Actin Dependent Regulator Of Chromatin, Subfamily A, Member 4	P51532	Protein Coding	50	GC19P010932	63.56
TGFB1	Transforming Growth Factor Beta 1	P01137	Protein Coding	52	GC19M041301	62.26
FGFR2	Fibroblast Growth Factor Receptor 2	P21802	Protein Coding	54	GC10M121478	61.28
MTOR	Mechanistic Target Of Rapamycin Kinase	P42345	Protein Coding	54	GC01M011106	61.02

NRAS	NRAS Proto-Oncogene, GTPase	P01111	Protein Coding	50	GC01M114704	59.22
DICER1	Dicer 1, Ribonuclease III	Q9UPY3	Protein Coding	47	GC14M095086	58.91
EPCAM	Epithelial Cell Adhesion Molecule	P16422	Protein Coding	47	GC02P047345	58.18
IL1RN	Interleukin 1 Receptor Antagonist	P18510	Protein Coding	48	GC02P115307	57.97
NOTCH1	Notch Receptor 1	P46531	Protein Coding	51	GC09M136602	57.63
CASP8	Caspase 8	Q14790	Protein Coding	52	GC02P201233	57.46
PTCH1	Patched 1	Q13635	Protein Coding	50	GC09M095442	57.44
BARD1	BRCA1 Associated RING Domain 1	Q99728	Protein Coding	46	GC02M214725	55.32
CDKN2B	Cyclin Dependent Kinase Inhibitor 2B	P42772	Protein Coding	47	GC09M022002	55.09
FASLG	Fas Ligand	P48023	Protein Coding	47	GC01P172628	53.98
RET	Ret Proto-Oncogene	P07949	Protein Coding	53	GC10P043081	53.91
MDM2	MDM2 Proto-Oncogene	Q00987	Protein Coding	52	GC12P068808	53.54
FGFR1	Fibroblast Growth Factor Receptor 1	P11362	Protein Coding	55	GC08M038400	52.82
IL10	Interleukin 10	P22301	Protein Coding	47	GC01M206767	52.12
IGF2	Insulin Like Growth Factor 2	P01344	Protein Coding	48	GC11M002130	52.1
MGMT	O-6-Methylguanine-DNA Methyltransferase	P16455	Protein Coding	50	GC10P129467	51.81
IRF1	Interferon Regulatory Factor 1	P10914	Protein Coding	47	GC05M132481	51.44
TYMP	Thymidine Phosphorylase	P19971	Protein Coding	46	GC22M050525	51.31
RAF1	Raf-1 Proto-Oncogene, Serine/Threonine Kinase	P04049	Protein Coding	54	GC03M012583	51.3
DNMT1	DNA Methyltransferase 1	P26358	Protein Coding	50	GC19M010133	51.09
MAP2K1	Mitogen-Activated Protein Kinase Kinase 1	Q02750	Protein Coding	54	GC15P066386	51.04
AURKA	Aurora Kinase A	O14965	Protein Coding	50	GC20M056370	50.84
PDGFRA	Platelet Derived Growth Factor Receptor Alpha	P16234	Protein Coding	55	GC04P054229	50.59
FHIT	Fragile Histidine Triad Diadenosine Triphosphatase	P49789	Protein Coding	44	GC03M059747	50.4
EZH2	Enhancer Of Zeste 2 Polycomb Repressive Complex 2 Subunit	Q15910	Protein Coding	54	GC07M148807	50.25
SMAD3	SMAD Family Member 3	P84022	Protein Coding	49	GC15P067063	50.23

PIK3R1	Phosphoinositide-3-Kinase Regulatory Subunit 1	P27986	Protein Coding	51	GC05P068215	50.23
TOP2A	DNA Topoisomerase II Alpha	P11388	Protein Coding	50	GC17M040388	49.72
PLAU	Plasminogen Activator, Urokinase	P00749	Protein Coding	51	GC10P073909	49.63
MTHFR	Methylenetetrahydrofolate Reductase	P42898	Protein Coding	47	GC01M011785	49.33
AKT2	AKT Serine/Threonine Kinase 2	P31751	Protein Coding	54	GC19M040230	49.31
TLR4	Toll Like Receptor 4	O00206	Protein Coding	51	GC09P117704	49.16
RAD51C	RAD51 Paralog C	O43502	Protein Coding	43	GC17P058692	49.1
MSH3	MutS Homolog 3	P20585	Protein Coding	43	GC05P080654	48.81
RAD51D	RAD51 Paralog D	O75771	Protein Coding	40	GC17M035092	48.3
BRIP1	BRCA1 Interacting Protein C-Terminal Helicase 1	Q9BX63	Protein Coding	47	GC17M061679	48.28
ESR2	Estrogen Receptor 2	Q92731	Protein Coding	49	GC14M064084	48.17
FLT4	Fms Related Receptor Tyrosine Kinase 4	P35916	Protein Coding	52	GC05M180607	47.6
MEN1	Menin 1	O00255	Protein Coding	46	GC11M064803	47.42
TGFBR1	Transforming Growth Factor Beta Receptor 1	P36897	Protein Coding	52	GC09P099104	47.3
POLE	DNA Polymerase Epsilon, Catalytic Subunit	Q07864	Protein Coding	48	GC12M132624	47.14
POLD1	DNA Polymerase Delta 1, Catalytic Subunit	P28340	Protein Coding	45	GC19P050385	47.14
SLC2A1	Solute Carrier Family 2 Member 1	P11166	Protein Coding	52	GC01M042925	46.96
AR	Androgen Receptor	P10275	Protein Coding	53	GC0XP067544	46.05
ERCC1	ERCC Excision Repair 1, Endonuclease Non-Catalytic Subunit	P07992	Protein Coding	45	GC19M045409	45.89
MRE11	MRE11 Homolog, Double Strand Break Repair Nuclease	P49959	Protein Coding	41	GC11M094444	45.84
SMARCB1	SWI/SNF Related, Matrix Associated, Actin Dependent Regulator Of Chromatin, Subfamily B, Member 1	Q12824	Protein Coding	45	GC22P023786	45.49
TCF7L2	Transcription Factor 7 Like 2	Q9NQB0	Protein Coding	45	GC10P112950	45.41
CXCR4	C-X-C Motif Chemokine Receptor 4	P61073	Protein Coding	52	GC02M136114	45.39
TWIST1	Twist Family BHLH Transcription Factor 1	Q15672	Protein Coding	45	GC07M019020	45.36
TSC2	TSC Complex Subunit 2	P49815	Protein Coding	50	GC16P002436	45.26

KLF6	Kruppel Like Factor 6	Q99612	Protein Coding	44 GC10M003779	45.16
SOX2	SRY-Box Transcription Factor 2	P48431	Protein Coding	47 GC03P181711	44.98
CASP10	Caspase 10	Q92851	Protein Coding	48 GC02P201182	44.81
GLI1	GLI Family Zinc Finger 1	P08151	Protein Coding	47 GC12P057460	44.66
SMAD7	SMAD Family Member 7	O15105	Protein Coding	43 GC18M048919	44.35
YAP1	Yes1 Associated Transcriptional Regulator	P46937	Protein Coding	47 GC11P102110	44.32
IFNG	Interferon Gamma	P01579	Protein Coding	48 GC12M068064	44.15
TIMP3	TIMP Metallopeptidase Inhibitor 3	P35625	Protein Coding	45 GC22P032800	43.69
PTGS2	Prostaglandin-Endoperoxide Synthase 2	P35354	Protein Coding	48 GC01M186640	43.41
TGFB2	Transforming Growth Factor Beta 2	P61812	Protein Coding	50 GC01P218345	43.39
INS	Insulin	P01308	Protein Coding	48 GC11M002159	43.27
NFE2L2	Nuclear Factor, Erythroid 2 Like 2	Q16236	Protein Coding	48 GC02M177227	42.98
RAD50	RAD50 Double Strand Break Repair Protein	Q92878	Protein Coding	49 GC05P132556	42.69
TP73	Tumor Protein P73	O15350	Protein Coding	45 GC01P003652	42.65
WNT5A	Wnt Family Member 5A	P41221	Protein Coding	49 GC03M055465	42.61
PPARG	Peroxisome Proliferator Activated Receptor Gamma	P37231	Protein Coding	52 GC03P012287	42.48
FH	Fumarate Hydratase	P07954	Protein Coding	45 GC01M241499	42.41
BCL2	BCL2 Apoptosis Regulator	P10415	Protein Coding	51 GC18M063123	42.31
PDGFRB	Platelet Derived Growth Factor Receptor Beta	P09619	Protein Coding	55 GC05M150113	41.76
PTPN11	Protein Tyrosine Phosphatase Non-Receptor Type 11	Q06124	Protein Coding	53 GC12P112418	41.66
AXIN1	Axin 1	O15169	Protein Coding	47 GC16M000287	41.44
MUC1	Mucin 1, Cell Surface Associated	P15941	Protein Coding	47 GC01M155185	41.04
ODC1	Ornithine Decarboxylase 1	P11926	Protein Coding	47 GC02M010432	41.04
NBN	Nibrin	O60934	Protein Coding	47 GC08M089933	40.93
CXCL12	C-X-C Motif Chemokine Ligand 12	P48061	Protein Coding	45 GC10M044294	40.84
CXCL8	C-X-C Motif Chemokine Ligand 8	P10145	Protein Coding	41 GC04P073740	40.74
RAD51	RAD51 Recombinase	Q06609	Protein Coding	52 GC15P040694	40.74

DDB2	Damage Specific DNA Binding Protein 2	Q92466	Protein Coding	45	GC11P047237	40.72
OGG1	8-Oxoguanine DNA Glycosylase	O15527	Protein Coding	47	GC03P009751	40.71
SRC	SRC Proto-Oncogene, Non-Receptor Tyrosine Kinase	P12931	Protein Coding	51	GC20P037344	40.61
SDHB	Succinate Dehydrogenase Complex Iron Sulfur Subunit B	P21912	Protein Coding	47	GC01M017020	40.55
STAT1	Signal Transducer And Activator Of Transcription 1	P42224	Protein Coding	53	GC02M190908	40.53
WNT1	Wnt Family Member 1	P04628	Protein Coding	46	GC12P049053	40.4
ARID1A	AT-Rich Interaction Domain 1A	O14497	Protein Coding	44	GC01P026693	40.4
FLT1	Fms Related Receptor Tyrosine Kinase 1	P17948	Protein Coding	51	GC13M028300	39.76
FGFR3	Fibroblast Growth Factor Receptor 3	P22607	Protein Coding	55	GC04P001795	39.44
CASP3	Caspase 3	P42574	Protein Coding	50	GC04M184627	39.13
MAPK1	Mitogen-Activated Protein Kinase 1	P28482	Protein Coding	51	GC22M021754	38.99
EP300	E1A Binding Protein P300	Q09472	Protein Coding	50	GC22P041091	38.6
ETS1	ETS Proto-Oncogene 1, Transcription Factor	P14921	Protein Coding	49	GC11M128458	38.44
GAST	Gastrin	P01350	Protein Coding	40	GC17P041712	38.4
DPYD	Dihydropyrimidine Dehydrogenase	Q12882	Protein Coding	51	GC01M097015	38.36
MMP9	Matrix Metallopeptidase 9	P14780	Protein Coding	52	GC20P046008	37.99
SPP1	Secreted Phosphoprotein 1	P10451	Protein Coding	46	GC04P087975	37.61
ABCB1	ATP Binding Cassette Subfamily B Member 1	P08183	Protein Coding	51	GC07M087504	37.47
NFKB1	Nuclear Factor Kappa B Subunit 1	P19838	Protein Coding	52	GC04P102501	37.3
JUN	Jun Proto-Oncogene, AP-1 Transcription Factor Subunit	P05412	Protein Coding	49	GC01M058780	37.24
HIF1A	Hypoxia Inducible Factor 1 Subunit Alpha	Q16665	Protein Coding	47	GC14P061695	36.97
TP63	Tumor Protein P63	Q9H3D4	Protein Coding	48	GC03P189598	36.97
ACTB	Actin Beta	P60709	Protein Coding	49	GC07M005527	36.88
ATP4A	ATPase H+/K+ Transporting Subunit Alpha	P20648	Protein Coding	41	GC19M042544	36.75
ZFHX3	Zinc Finger Homeobox 3	Q15911	Protein Coding	41	GC16M072782	36.73
MMP2	Matrix Metallopeptidase 2	P08253	Protein Coding	53	GC16P055390	36.62

PROM1	Prominin 1	O43490	Protein Coding	44	GC04M015965	36.62
TFF1	Trefoil Factor 1	P04155	Protein Coding	44	GC21M042362	36.51
GSTP1	Glutathione S-Transferase Pi 1	P09211	Protein Coding	50	GC11P067583	36.51
TSC1	TSC Complex Subunit 1	Q92574	Protein Coding	48	GC09M132891	36.43
ERCC2	ERCC Excision Repair 2, TFIIH Core Complex Helicase Subunit	P18074	Protein Coding	47	GC19M045349	36.22
XIAP	X-Linked Inhibitor Of Apoptosis	P98170	Protein Coding	49	GC0XP123859	36.11
NOTCH3	Notch Receptor 3	Q9UM47	Protein Coding	49	GC19M015159	36.02
HSPB1	Heat Shock Protein Family B (Small) Member 1	P04792	Protein Coding	51	GC07P076302	36.01
ZEB2	Zinc Finger E-Box Binding Homeobox 2	O60315	Protein Coding	48	GC02M144384	35.94
JAK2	Janus Kinase 2	O60674	Protein Coding	54	GC09P004985	35.81
MUC5AC	Mucin 5AC, Oligomeric Mucus/Gel-Forming	P98088	Protein Coding	39	GC11P001151	35.8
GSTM1	Glutathione S-Transferase Mu 1	P09488	Protein Coding	41	GC01P109687	35.72
PSCA	Prostate Stem Cell Antigen	O43653	Protein Coding	39	GC08P142670	35.47
ZEB1	Zinc Finger E-Box Binding Homeobox 1	P37275	Protein Coding	48	GC10P031318	35.38
IGF1R	Insulin Like Growth Factor 1 Receptor	P08069	Protein Coding	54	GC15P098648	35.33
FGF2	Fibroblast Growth Factor 2	P09038	Protein Coding	47	GC04P122826	35.28
ERCC6	ERCC Excision Repair 6, Chromatin Remodeling Factor	Q03468	Protein Coding	45	GC10M049454	35.21
CD274	CD274 Molecule	Q9NZQ7	Protein Coding	44	GC09P005450	35.2
JAG1	Jagged Canonical Notch Ligand 1	P78504	Protein Coding	50	GC20M010637	35.14
PDCD1	Programmed Cell Death 1	Q15116	Protein Coding	48	GC02M241849	34.83
ERBB4	Erb-B2 Receptor Tyrosine Kinase 4	Q15303	Protein Coding	55	GC02M211375	34.77
HGF	Hepatocyte Growth Factor	P14210	Protein Coding	52	GC07M081699	34.68
BIRC5	Baculoviral IAP Repeat Containing 5	O15392	Protein Coding	47	GC17P078214	34.65
IGF1	Insulin Like Growth Factor 1	P05019	Protein Coding	50	GC12M102395	34.32
CD40LG	CD40 Ligand	P29965	Protein Coding	47	GC0XP136649	34.31
NOS2	Nitric Oxide Synthase 2	P35228	Protein Coding	49	GC17M027756	34.22

LEP	Leptin	P41159	Protein Coding	47	GC07P128241	34.2
CD44	CD44 Molecule (Indian Blood Group)	P16070	Protein Coding	47	GC11P035139	34.15
HMOX1	Heme Oxygenase 1	P09601	Protein Coding	52	GC22P035380	34.05
TGFA	Transforming Growth Factor Alpha	P01135	Protein Coding	46	GC02M070447	33.78
NF2	Neurofibromin 2	P35240	Protein Coding	48	GC22P029603	33.7
NOTCH2	Notch Receptor 2	Q04721	Protein Coding	50	GC01M119911	33.67
CAT	Catalase	P04040	Protein Coding	50	GC11P034460	33.65
CEACAM5	CEA Cell Adhesion Molecule 5	P06731	Protein Coding	41	GC19P041709	33.6
IDH1	Isocitrate Dehydrogenase (NADP(+)) 1	O75874	Protein Coding	52	GC02M208236	33.56
VHL	Von Hippel-Lindau Tumor Suppressor	P40337	Protein Coding	47	GC03P010211	33.52
PRKAR1A	Protein Kinase CAMP-Dependent Type I Regulatory Subunit Alpha	P10644	Protein Coding	51	GC17P068414	33.51
RELA	RELA Proto-Oncogene, NF-KB Subunit	Q04206	Protein Coding	50	GC11M065653	33.42
MAP2K2	Mitogen-Activated Protein Kinase Kinase 2	P36507	Protein Coding	53	GC19M004090	33.39
TACC1	Transforming Acidic Coiled-Coil Containing Protein 1	O75410	Protein Coding	40	GC08P038728	33.35
BCL2L1	BCL2 Like 1	Q07817	Protein Coding	47	GC20M031664	33.2
HNF4A	Hepatocyte Nuclear Factor 4 Alpha	P41235	Protein Coding	50	GC20P044355	33.14
MUC6	Mucin 6, Oligomeric Mucus/Gel-Forming	Q6W4X9	Protein Coding	37	GC11M001002	33.13
TNFRSF10B	TNF Receptor Superfamily Member 10b	O14763	Protein Coding	50	GC08M023020	32.95
MAPK3	Mitogen-Activated Protein Kinase 3	P27361	Protein Coding	49	GC16M030117	32.9
CDX2	Caudal Type Homeobox 2	Q99626	Protein Coding	43	GC13M027962	32.69
CDKN3	Cyclin Dependent Kinase Inhibitor 3	Q16667	Protein Coding	42	GC14P054398	32.61
TYMS	Thymidylate Synthetase	P04818	Protein Coding	47	GC18P000657	32.55
WNT2B	Wnt Family Member 2B	Q93097	Protein Coding	44	GC01P112466	32.55
CREB1	CAMP Responsive Element Binding Protein 1	P16220	Protein Coding	48	GC02P207529	32.55
GHRL	Ghrelin And Obestatin Prepropeptide	Q9UBU3	Protein Coding	43	GC03M010285	32.36
CHEK1	Checkpoint Kinase 1	O14757	Protein Coding	50	GC11P125625	32.03

FOXP3	Forkhead Box P3	Q9BZS1	Protein Coding	46	GC0XM049250	31.95
BIRC3	Baculoviral IAP Repeat Containing 3	Q13489	Protein Coding	46	GC11P102317	31.91
NKX2-1	NK2 Homeobox 1	P43699	Protein Coding	46	GC14M036516	31.71
SNAI2	Snail Family Transcriptional Repressor 2	O43623	Protein Coding	44	GC08M048854	31.54
SDHD	Succinate Dehydrogenase Complex Subunit D	O14521	Protein Coding	44	GC11P112087	31.48
XRCC1	X-Ray Repair Cross Complementing 1	P18887	Protein Coding	43	GC19M043543	31.37
NTRK1	Neurotrophic Receptor Tyrosine Kinase 1	P04629	Protein Coding	48	GC01P156786	31.3
NFKBIA	NFKB Inhibitor Alpha	P25963	Protein Coding	50	GC14M035401	31.25
BAP1	BRCA1 Associated Protein 1	Q92560	Protein Coding	45	GC03M052401	31.23
SDHA	Succinate Dehydrogenase Complex Flavoprotein Subunit A	P31040	Protein Coding	46	GC05P000208	31.05
LRP5	LDL Receptor Related Protein 5	O75197	Protein Coding	48	GC11P068298	30.99
VEGFC	Vascular Endothelial Growth Factor C	P49767	Protein Coding	47	GC04M176683	30.97
SDHC	Succinate Dehydrogenase Complex Subunit C	Q99643	Protein Coding	44	GC01P161314	30.96
RASSF1	Ras Association Domain Family Member 1	Q9NS23	Protein Coding	44	GC03M050329	30.7
CDK2	Cyclin Dependent Kinase 2	P24941	Protein Coding	52	GC12P055966	30.58
PLAUR	Plasminogen Activator, Urokinase Receptor	Q03405	Protein Coding	44	GC19M043646	30.56
BCL10	BCL10 Immune Signaling Adaptor	O95999	Protein Coding	45	GC01M085265	30.54
KDR	Kinase Insert Domain Receptor	P35968	Protein Coding	53	GC04M055078	30.51
SP1	Sp1 Transcription Factor	P08047	Protein Coding	44	GC12P053380	30.33
E2F1	E2F Transcription Factor 1	Q01094	Protein Coding	43	GC20M033675	30.23
SST	Somatostatin	P61278	Protein Coding	42	GC03M187668	30.19
NQO1	NAD(P)H Quinone Dehydrogenase 1	P15559	Protein Coding	49	GC16M069706	30.11
CASP9	Caspase 9	P55211	Protein Coding	48	GC01M015491	30.05
CCNE1	Cyclin E1	P24864	Protein Coding	48	GC19P029811	30.04
CDK6	Cyclin Dependent Kinase 6	Q00534	Protein Coding	54	GC07M092604	29.94
WT1	WT1 Transcription Factor	P19544	Protein Coding	49	GC11M032365	29.82

PGR	Progesterone Receptor	P06401	Protein Coding	50	GC11M100943	29.64
ERBB3	Erb-B2 Receptor Tyrosine Kinase 3	P21860	Protein Coding	54	GC12P056094	29.61
CDH3	Cadherin 3	P22223	Protein Coding	47	GC16P068637	29.6
RARB	Retinoic Acid Receptor Beta	P10826	Protein Coding	50	GC03P024830	29.54
KRT20	Keratin 20	P35900	Protein Coding	41	GC17M040875	29.47
ICAM1	Intercellular Adhesion Molecule 1	P05362	Protein Coding	50	GC19P010270	29.4
TLR2	Toll Like Receptor 2	O60603	Protein Coding	51	GC04P153684	29.39
ALB	Albumin	P02768	Protein Coding	50	GC04P073397	29.39
BMP2	Bone Morphogenetic Protein 2	P12643	Protein Coding	47	GC20P006696	29.33
ABCG2	ATP Binding Cassette Subfamily G Member 2 (Junior Blood Group)	Q9UNQ0	Protein Coding	50	GC04M088090	29.11
RPS6KB1	Ribosomal Protein S6 Kinase B1	P23443	Protein Coding	49	GC17P059893	29
MMP7	Matrix Metallopeptidase 7	P09237	Protein Coding	48	GC11M102425	28.94
FANCC	FA Complementation Group C	Q00597	Protein Coding	47	GC09M095099	28.87
CTLA4	Cytotoxic T-Lymphocyte Associated Protein 4	P16410	Protein Coding	45	GC02P203867	28.85
SMAD2	SMAD Family Member 2	Q15796	Protein Coding	47	GC18M047809	28.8
DLEC1	DLEC1 Cilia And Flagella Associated Protein	Q9Y238	Protein Coding	36	GC03P038038	28.8
PDGFB	Platelet Derived Growth Factor Subunit B	P01127	Protein Coding	50	GC22M045657	28.78
ENG	Endoglin	P17813	Protein Coding	46	GC09M127815	28.73
CYCS	Cytochrome C, Somatic	P99999	Protein Coding	48	GC07M025118	28.64
WWOX	WW Domain Containing Oxidoreductase	Q9NZC7	Protein Coding	47	GC16P078099	28.34
MAPK8	Mitogen-Activated Protein Kinase 8	P45983	Protein Coding	50	GC10P048306	28.12
CD40	CD40 Molecule	P25942	Protein Coding	48	GC20P046118	28.08
AFP	Alpha Fetoprotein	P02771	Protein Coding	45	GC04P073431	27.96
PRKCA	Protein Kinase C Alpha	P17252	Protein Coding	50	GC17P066302	27.87
H3-3A	H3.3 Histone A	P84243	Protein Coding	34	GC01P226062	27.86
BAK1	BCL2 Antagonist/Killer 1	Q16611	Protein Coding	44	GC06M033572	27.79

FOS	Fos Proto-Oncogene, AP-1 Transcription Factor Subunit	P01100	Protein Coding	50 GC14P075278	27.78
SNAI1	Snail Family Transcriptional Repressor 1	O95863	Protein Coding	44 GC20P049982	27.71
SOD2	Superoxide Dismutase 2	P04179	Protein Coding	51 GC06M159669	27.61
ABCC1	ATP Binding Cassette Subfamily C Member 1	P33527	Protein Coding	47 GC16P015949	27.51
PCNA	Proliferating Cell Nuclear Antigen	P12004	Protein Coding	51 GC20M005114	27.46
SUFU	SUFU Negative Regulator Of Hedgehog Signaling	Q9UMX1	Protein Coding	43 GC10P102503	27.44
B2M	Beta-2-Microglobulin	P61769	Protein Coding	48 GC15P044711	27.35
ABL1	ABL Proto-Oncogene 1, Non-Receptor Tyrosine Kinase	P00519	Protein Coding	52 GC09P130713	27.31
RHOA	Ras Homolog Family Member A	P61586	Protein Coding	46 GC03M049359	27.3
KRT19	Keratin 19	P08727	Protein Coding	45 GC17M041523	27.19
CCKBR	Cholecystokinin B Receptor	P32239	Protein Coding	44 GC11P006259	27.16
ROS1	ROS Proto-Oncogene 1, Receptor Tyrosine Kinase	P08922	Protein Coding	45 GC06M117287	27.13
MPO	Myeloperoxidase	P05164	Protein Coding	50 GC17M058269	27.12
U2AF1	U2 Small Nuclear RNA Auxiliary Factor 1	Q01081	Protein Coding	41 GC21M043092	27.03
PIK3CB	Phosphatidylinositol-4,5-Bisphosphate 3-Kinase Catalytic Subunit Beta	P42338	Protein Coding	47 GC03M138652	26.94
LBR	Lamin B Receptor	Q14739	Protein Coding	47 GC01M225401	26.88
PHB	Prohibitin	P35232	Protein Coding	47 GC17M049404	26.86
SHC1	SHC Adaptor Protein 1	P29353	Protein Coding	45 GC01M154962	26.85
PLK1	Polo Like Kinase 1	P53350	Protein Coding	49 GC16P023888	26.84
E2F3	E2F Transcription Factor 3	O00716	Protein Coding	43 GC06P020402	26.82
GSK3B	Glycogen Synthase Kinase 3 Beta	P49841	Protein Coding	50 GC03M119821	26.82
RUNX3	RUNX Family Transcription Factor 3	Q13761	Protein Coding	43 GC01M024899	26.72
BLM	BLM RecQ Like Helicase	P54132	Protein Coding	48 GC15P090717	26.71
PMS1	PMS1 Homolog 1, Mismatch Repair System Component	P54277	Protein Coding	40 GC02P189784	26.69
CCNB1	Cyclin B1	P14635	Protein Coding	47 GC05P069167	26.64
S100A8	S100 Calcium Binding Protein A8	P05109	Protein Coding	42 GC01M153391	26.64

TIMP1	TIMP Metallopeptidase Inhibitor 1	P01033	Protein Coding	45 GC0XP047583	26.62
MAP3K6	Mitogen-Activated Protein Kinase Kinase Kinase 6	O95382	Protein Coding	44 GC01M027365	26.56
MMP14	Matrix Metallopeptidase 14	P50281	Protein Coding	51 GC14P025277	26.54
SERPINA3	Serpin Family A Member 3	P01011	Protein Coding	43 GC14P094612	26.5
RNF43	Ring Finger Protein 43	Q68DV7	Protein Coding	37 GC17M058352	26.5
MUC2	Mucin 2, Oligomeric Mucus/Gel-Forming	Q02817	Protein Coding	38 GC11P001074	26.46
IL2	Interleukin 2	P60568	Protein Coding	45 GC04M122451	26.38
MCM4	Minichromosome Maintenance Complex Component 4	P33991	Protein Coding	47 GC08P047965	26.36
DNMT3A	DNA Methyltransferase 3 Alpha	Q9Y6K1	Protein Coding	51 GC02M025228	26.28
NME1	NME/NM23 Nucleoside Diphosphate Kinase 1	P15531	Protein Coding	47 GC17P051154	26.23
MYCN	MYCN Proto-Oncogene, BHLH Transcription Factor	P04198	Protein Coding	46 GC02P015949	26.16
CYP17A1	Cytochrome P450 Family 17 Subfamily A Member 1	P05093	Protein Coding	48 GC10M102830	26.16
ALK	ALK Receptor Tyrosine Kinase	Q9UM73	Protein Coding	51 GC02M029156	26.13
ABCC2	ATP Binding Cassette Subfamily C Member 2	Q92887	Protein Coding	47 GC10P099782	26.05
ITGB1	Integrin Subunit Beta 1	P05556	Protein Coding	50 GC10M032900	26
TIMP2	TIMP Metallopeptidase Inhibitor 2	P16035	Protein Coding	44 GC17M078852	25.95
THBS1	Thrombospondin 1	P07996	Protein Coding	44 GC15P039581	25.92
HRH2	Histamine Receptor H2	P25021	Protein Coding	44 GC05P175659	25.91
UBE2T	Ubiquitin Conjugating Enzyme E2 T	Q9NPD8	Protein Coding	43 GC01M202300	25.88
RPL15	Ribosomal Protein L15	P61313	Protein Coding	44 GC03P023960	25.84
SHH	Sonic Hedgehog Signaling Molecule	Q15465	Protein Coding	50 GC07M155799	25.83
CYP2D6	Cytochrome P450 Family 2 Subfamily D Member 6	P10635	Protein Coding	48 GC22M042126	25.79
BAD	BCL2 Associated Agonist Of Cell Death	Q92934	Protein Coding	46 GC11M064273	25.78
FLNC	Filamin C	Q14315	Protein Coding	44 GC07P128830	25.78
GRB2	Growth Factor Receptor Bound Protein 2	P62993	Protein Coding	49 GC17M075318	25.75
CHGA	Chromogranin A	P10645	Protein Coding	42 GC14P092923	25.71

KRT7	Keratin 7	P08729	Protein Coding	41	GC12P052232	25.65
ADIPOQ	Adiponectin, C1Q And Collagen Domain Containing	Q15848	Protein Coding	45	GC03P186842	25.62
FZD5	Frizzled Class Receptor 5	Q13467	Protein Coding	47	GC02M207762	25.58
PIK3R3	Phosphoinositide-3-Kinase Regulatory Subunit 3	Q92569	Protein Coding	43	GC01M046041	25.55
AREG	Amphiregulin	P15514	Protein Coding	43	GC04P074445	25.37
DNMT3B	DNA Methyltransferase 3 Beta	Q9UBC3	Protein Coding	50	GC20P032762	25.14
ANXA5	Annexin A5	P08758	Protein Coding	46	GC04M121667	25.13
F2	Coagulation Factor II, Thrombin	P00734	Protein Coding	48	GC11P046720	25.11
MTAP	Methylthioadenosine Phosphorylase	Q13126	Protein Coding	47	GC09P021792	24.98
PTK2	Protein Tyrosine Kinase 2	Q05397	Protein Coding	47	GC08M140657	24.94
KRT18	Keratin 18	P05783	Protein Coding	48	GC12P052948	24.9
LEF1	Lymphoid Enhancer Binding Factor 1	Q9UJU2	Protein Coding	47	GC04M108047	24.87
E2F2	E2F Transcription Factor 2	Q14209	Protein Coding	42	GC01M023527	24.79
FGF1	Fibroblast Growth Factor 1	P05230	Protein Coding	48	GC05M142555	24.79
MMP1	Matrix Metallopeptidase 1	P03956	Protein Coding	51	GC11M102810	24.74
CCND2	Cyclin D2	P30279	Protein Coding	50	GC12P008103	24.69
NCOA3	Nuclear Receptor Coactivator 3	Q9Y6Q9	Protein Coding	45	GC20P047501	24.63
KITLG	KIT Ligand	P21583	Protein Coding	44	GC12M088492	24.6
FEZF1	FEZ Family Zinc Finger 1	A0PJY2	Protein Coding	39	GC07M122301	24.57
ACE	Angiotensin I Converting Enzyme	P12821	Protein Coding	49	GC17P063477	24.55
TFF2	Trefoil Factor 2	Q03403	Protein Coding	41	GC21M042346	24.44
CTSD	Cathepsin D	P07339	Protein Coding	52	GC11M001752	24.4
PRKCD	Protein Kinase C Delta	Q05655	Protein Coding	53	GC03P053156	24.33
CRP	C-Reactive Protein	P02741	Protein Coding	46	GC01M159716	24.13
HLA-DRB1	Major Histocompatibility Complex, Class II, DR Beta 1	P01911	Protein Coding	46	GC06M032578	24.1
CYP1A1	Cytochrome P450 Family 1 Subfamily A Member 1	P04798	Protein Coding	47	GC15M074719	24.1
IDH2	Isocitrate Dehydrogenase (NADP(+)) 2	P48735	Protein Coding	52	GC15M090083	24.08

VEGFD	Vascular Endothelial Growth Factor D	O43915	Protein Coding	34	GC0XM01534 5	24.08
WRN	WRN RecQ Like Helicase	Q14191	Protein Coding	45	GC08P031033	24.08
DAPK1	Death Associated Protein Kinase 1	P53355	Protein Coding	48	GC09P087497	24.06
MUC4	Mucin 4, Cell Surface Associated	Q99102	Protein Coding	38	GC03M195746	24.01
GATA3	GATA Binding Protein 3	P23771	Protein Coding	49	GC10P008045	24
TNFSF10	TNF Superfamily Member 10	P50591	Protein Coding	46	GC03M172505	23.99
FGF7	Fibroblast Growth Factor 7	P21781	Protein Coding	41	GC15P049423	23.97
CFLAR	CASP8 And FADD Like Apoptosis Regulator	O15519	Protein Coding	46	GC02P201117	23.94
JAK3	Janus Kinase 3	P52333	Protein Coding	51	GC19M017824	23.93
EDNRA	Endothelin Receptor Type A	P25101	Protein Coding	49	GC04P147480	23.83
MMP3	Matrix Metallopeptidase 3	P08254	Protein Coding	51	GC11M102835	23.76
CDK1	Cyclin Dependent Kinase 1	P06493	Protein Coding	45	GC10P060772	23.74
CBLIF	Cobalamin Binding Intrinsic Factor	P27352	Protein Coding	34	GC11M059829	23.74
IGFBP3	Insulin Like Growth Factor Binding Protein 3	P17936	Protein Coding	45	GC07M045912	23.67
CCR6	C-C Motif Chemokine Receptor 6	P51684	Protein Coding	44	GC06P167111	23.67
HNF1B	HNF1 Homeobox B	P35680	Protein Coding	44	GC17M037686	23.64
TTN	Titin	Q8WZ42	Protein Coding	47	GC02M178525	23.63
PAX5	Paired Box 5	Q02548	Protein Coding	46	GC09M036828	23.58
HLA-A	Major Histocompatibility Complex, Class I, A	P04439	Protein Coding	46	GC06P047265	23.56
CDH2	Cadherin 2	P19022	Protein Coding	50	GC18M027950	23.54
JUP	Junction Plakoglobin	P14923	Protein Coding	47	GC17M041754	23.46
POMC	Proopiomelanocortin	P01189	Protein Coding	48	GC02M025160	23.46
SERPINB5	Serpin Family B Member 5	P36952	Protein Coding	43	GC18P063476	23.43
IL7	Interleukin 7	P13232	Protein Coding	42	GC08M078689	23.42
HBEGF	Heparin Binding EGF Like Growth Factor	Q99075	Protein Coding	43	GC05M140332	23.41
COMT	Catechol-O-Methyltransferase	P21964	Protein Coding	51	GC22P019941	23.4
PTGS1	Prostaglandin-Endoperoxide Synthase 1	P23219	Protein Coding	46	GC09P122370	23.37

MCL1	MCL1 Apoptosis Regulator, BCL2 Family Member	Q07820	Protein Coding	47	GC01M150707	23.36
SKP2	S-Phase Kinase Associated Protein 2	Q13309	Protein Coding	44	GC05P036103	23.23
SOX4	SRY-Box Transcription Factor 4	Q06945	Protein Coding	43	GC06P021593	23.22
GADD45A	Growth Arrest And DNA Damage Inducible Alpha	P24522	Protein Coding	44	GC01P067685	23.2
CREBBP	CREB Binding Protein	Q92793	Protein Coding	52	GC16M003726	23.18
ATRX	ATRX Chromatin Remodeler	P46100	Protein Coding	45	GC0XM077504	23.09
WNT6	Wnt Family Member 6	Q9Y6F9	Protein Coding	41	GC02P218859	23.06
MAX	MYC Associated Factor X	P61244	Protein Coding	48	GC14M065009	23.06
PLCG1	Phospholipase C Gamma 1	P19174	Protein Coding	47	GC20P041136	23.05
IL17A	Interleukin 17A	Q16552	Protein Coding	42	GC06P052186	23.05
SERPINA1	Serpin Family A Member 1	P01009	Protein Coding	49	GC14M094376	23.01
CDKN1C	Cyclin Dependent Kinase Inhibitor 1C	P49918	Protein Coding	47	GC11M002887	22.87
GRP	Gastrin Releasing Peptide	P07492	Protein Coding	41	GC18P059220	22.83
NTRK3	Neurotrophic Receptor Tyrosine Kinase 3	Q16288	Protein Coding	51	GC15M087859	22.78
PAK1	P21 (RAC1) Activated Kinase 1	Q13153	Protein Coding	47	GC11M077321	22.76
SOX9	SRY-Box Transcription Factor 9	P48436	Protein Coding	47	GC17P072121	22.7
EPOR	Erythropoietin Receptor	P19235	Protein Coding	48	GC19M011377	22.67
CTAG2	Cancer/Testis Antigen 2	O75638	Protein Coding	33	GC0XM154651	22.58
NTHL1	Nth Like DNA Glycosylase 1	P78549	Protein Coding	44	GC16M002192	22.44
S100A4	S100 Calcium Binding Protein A4	P26447	Protein Coding	44	GC01M153543	22.44
SPINK1	Serine Peptidase Inhibitor Kazal Type 1	P00995	Protein Coding	43	GC05M147825	22.35
MAGEA1	MAGE Family Member A1	P43355	Protein Coding	37	GC0XP153179	22.33
EIF4EBP1	Eukaryotic Translation Initiation Factor 4E Binding Protein 1	Q13541	Protein Coding	47	GC08P038007	22.31
CD82	CD82 Molecule	P27701	Protein Coding	43	GC11P044586	22.29
CCNA2	Cyclin A2	P20248	Protein Coding	44	GC04M121816	22.27
IL4	Interleukin 4	P05112	Protein Coding	46	GC05P132673	22.26

KEAP1	Kelch Like ECH Associated Protein 1	Q14145	Protein Coding	48	GC19M010486	22.25
ALDH2	Aldehyde Dehydrogenase 2 Family Member	P05091	Protein Coding	50	GC12P111766	22.22
WNT3	Wnt Family Member 3	P56703	Protein Coding	46	GC17M046762	22.17
MAPK14	Mitogen-Activated Protein Kinase 14	Q16539	Protein Coding	51	GC06P047451	22.15
STAT5B	Signal Transducer And Activator Of Transcription 5B	P51692	Protein Coding	49	GC17M042199	22.12
DCC	DCC Netrin 1 Receptor	P43146	Protein Coding	46	GC18P052340	22.04
CCNE2	Cyclin E2	O96020	Protein Coding	41	GC08M094879	22.01
CSF3	Colony Stimulating Factor 3	P09919	Protein Coding	40	GC17P040015	22
CSF2	Colony Stimulating Factor 2	P04141	Protein Coding	44	GC05P132073	21.92
CTNND1	Catenin Delta 1	O60716	Protein Coding	45	GC11P057788	21.87
KRT17	Keratin 17	Q04695	Protein Coding	45	GC17M041619	21.85
CCK	Cholecystokinin	P06307	Protein Coding	41	GC03M042274	21.82
CASC2	Cancer Susceptibility 2	Q6XLA1	RNA	25	GC10P118046	21.8
MKI67	Marker Of Proliferation Ki-67	P46013	Protein Coding	44	GC10M128096	21.79
IKBKG	Inhibitor Of Nuclear Factor Kappa B Kinase Regulatory Subunit Gamma	Q9Y6K9	Protein Coding	48	GC0XP154541	21.74
WNT3A	Wnt Family Member 3A	P56704	Protein Coding	47	GC01P228127	21.72
H2AC18	H2A Clustered Histone 18	Q6FI13	Protein Coding	26	GC01M149961	21.67
FLCN	Folliculin	Q8NFG4	Protein Coding	41	GC17M017206	21.62
FGF4	Fibroblast Growth Factor 4	P08620	Protein Coding	44	GC11M069762	21.58
ECT2	Epithelial Cell Transforming 2	Q9H8V3	Protein Coding	40	GC03P172750	21.53
CD19	CD19 Molecule	P15391	Protein Coding	49	GC16P029083	21.52
ALOX5	Arachidonate 5-Lipoxygenase	P09917	Protein Coding	48	GC10P045374	21.51
CAV1	Caveolin 1	Q03135	Protein Coding	48	GC07P116524	21.39
MAGEA3	MAGE Family Member A3	P43357	Protein Coding	36	GC0XP152698	21.37
CALCA	Calcitonin Related Polypeptide Alpha	P06881	Protein Coding	43	GC11M014945	21.33
GKN1	Gastrokine 1	Q9NS71	Protein Coding	38	GC02P068974	21.31
TNFSF11	TNF Superfamily Member 11	O14788	Protein Coding	47	GC13P042562	21.29

MTDH	Metadherin	Q86UE4	Protein Coding	40	GC08P097643	21.1
SYP	Synaptophysin	P08247	Protein Coding	43	GC0XM049187	21.04
MUC16	Mucin 16, Cell Surface Associated	Q8WXI7	Protein Coding	36	GC19M008848	20.98
DES	Desmin	P17661	Protein Coding	48	GC02P219418	20.84
RXRA	Retinoid X Receptor Alpha	P19793	Protein Coding	49	GC09P134317	20.83
SOS2	SOS Ras/Rho Guanine Nucleotide Exchange Factor 2	Q07890	Protein Coding	45	GC14M050117	20.83
HSP90AA1	Heat Shock Protein 90 Alpha Family Class A Member 1	P07900	Protein Coding	48	GC14M102080	20.81
TMEM127	Transmembrane Protein 127	O75204	Protein Coding	37	GC02M096248	20.76
EPHA2	EPH Receptor A2	P29317	Protein Coding	52	GC01M016124	20.74
S100A6	S100 Calcium Binding Protein A6	P06703	Protein Coding	43	GC01M153535	20.72
NOD2	Nucleotide Binding Oligomerization Domain Containing 2	Q9HC29	Protein Coding	48	GC16P050693	20.71
GREM1	Gremlin 1, DAN Family BMP Antagonist	O60565	Protein Coding	44	GC15P032720	20.71
ENO2	Enolase 2	P09104	Protein Coding	47	GC12P006913	20.7
FZD7	Frizzled Class Receptor 7	O75084	Protein Coding	44	GC02P202034	20.68
AKT3	AKT Serine/Threonine Kinase 3	Q9Y243	Protein Coding	52	GC01M243488	20.63
BSG	Basigin (Ok Blood Group)	P35613	Protein Coding	44	GC19P000571	20.62
MYD88	MYD88 Innate Immune Signal Transduction Adaptor	Q99836	Protein Coding	50	GC03P038179	20.61
BTK	Bruton Tyrosine Kinase	Q06187	Protein Coding	53	GC0XM101349	20.57
CD34	CD34 Molecule	P28906	Protein Coding	43	GC01M207880	20.54
GKN2	Gastrokine 2	Q86XP6	Protein Coding	35	GC02M068945	20.42
KLF4	Kruppel Like Factor 4	O43474	Protein Coding	45	GC09M107484	20.42
BMI1	BMI1 Proto-Oncogene, Polycomb Ring Finger	P35226	Protein Coding	43	GC10P022326	20.38
CLDN4	Claudin 4	O14493	Protein Coding	41	GC07P073799	20.33
FBXW7	F-Box And WD Repeat Domain Containing 7	Q969H0	Protein Coding	44	GC04M152321	20.27
VIM	Vimentin	P08670	Protein Coding	50	GC10P017227	20.21
MALT1	MALT1 Paracaspase	Q9UDY8	Protein Coding	47	GC18P058671	20.17

RUNX2	RUNX Family Transcription Factor 2	Q13950	Protein Coding	47	GC06P047549	20.17
CYP19A1	Cytochrome P450 Family 19 Subfamily A Member 1	P11511	Protein Coding	48	GC15M051208	20.13
URGCP	Upregulator Of Cell Proliferation	Q8TCY9	Protein Coding	34	GC07M043876	20.1
WNT2	Wnt Family Member 2	P09544	Protein Coding	44	GC07M117276	20.1
