# Supplementary material for: An Investigation of the Antigastric Cancer Effect in Tumor Microenvironment of Radix Rhei Et Rhizome: A Network Pharmacology Study
Source: Evid Based Complement Alternat Med. 2021 Jun 24;2021:9913952. doi: 10.1155/2021/9913952 (PMC8249119; doi:10.1155/2021/9913952)
Supplement: Supplementary Materials. — Supplementary Table S1. GC-related targets. Supplementary Table S2. KEGG pathway analysis. [file 9913952.f1.zip › 9913952.f1/Supplementary Table S2. KEGG pathway analysis.pdf]

Supplementary Table S2. KEGG pathway analysis

Term	Count	%	PValue	Genes	List Total	Pop Hits	Pop Total
hsa04915:Estrogen signaling pathway	7	35	1.78E-07	HSP90AA1, PIK3CA, MMP2, ESR1, MMP9, EGFR, ESR2	20	99	6879
hsa05200:Pathways in cancer	10	50	3.28E-07	GSK3B, HSP90AA1, PIK3CA, CASP3, MMP2, CDK2, PTGS2, MET, MMP9, EGFR	20	393	6879
hsa05205:Proteoglycans in cancer	7	35	1.11E-05	PIK3CA, CASP3, MMP2, ESR1, MET, MMP9, EGFR	20	200	6879
hsa04115:p53 signaling pathway	5	25	2.85E-05	CCNB1, CASP3, IGFBP3, CDK2, CDK1	20	67	6879
hsa04914:Progesterone-mediated oocyte maturation	5	25	8.00E-05	HSP90AA1, CCNB1, PIK3CA, CDK2, CDK1	20	87	6879
hsa05215:Prostate cancer	5	25	8.37E-05	GSK3B, HSP90AA1, PIK3CA, CDK2, EGFR	20	88	6879
hsa04151:PI3K-Akt signaling pathway	7	35	2.37E-04	GSK3B, HSP90AA1, FLT1, PIK3CA, CDK2, MET, EGFR	20	345	6879
hsa05206:MicroRNAs in cancer	6	30	8.62E-04	ABCB1, CASP3, PTGS2, MET, MMP9, EGFR	20	286	6879
hsa04917:Prolactin signaling pathway	4	20	9.07E-04	GSK3B, PIK3CA, ESR1, ESR2	20	71	6879
hsa04510:Focal adhesion	5	25	0.002125	GSK3B, FLT1, PIK3CA, MET, EGFR	20	206	6879
hsa04668:TNF signaling pathway	4	20	0.002958	PIK3CA, CASP3, PTGS2, MMP9	20	107	6879
hsa04110:Cell cycle	4	20	0.004486	GSK3B, CCNB1, CDK2, CDK1	20	124	6879
hsa05219:Bladder cancer	3	15	0.005559	MMP2, MMP9, EGFR	20	41	6879
hsa04068:FoxO signaling pathway	4	20	0.005573	CCNB1, PIK3CA, CDK2, EGFR	20	134	6879
hsa05161:Hepatitis B	4	20	0.006939	PIK3CA, CASP3, CDK2, MMP9	20	145	6879
hsa05213:Endometrial cancer	3	15	0.008828	GSK3B, PIK3CA, EGFR	20	52	6879
hsa04923:Regulation of lipolysis in adipocytes	3	15	0.010185	PIK3CA, PTGS2, PTGS1	20	56	6879

hsa05202:Transcriptional misregulation in cancer	4	20	0.01023	FLT1, IGFBP3, MET, MMP9	20	167	6879
hsa05210:Colorectal cancer	3	15	0.012385	GSK3B, PIK3CA, CASP3	20	62	6879
hsa05230:Central carbon metabolism in cancer	3	15	0.01316	PIK3CA, MET, EGFR	20	64	6879
hsa05120:Epithelial cell signaling in Helicobacter pylori	3	15	0.014362	CASP3, MET, EGFR	20	67	6879
hsa05218:Melanoma	3	15	0.016036	PIK3CA, MET, EGFR	20	71	6879
hsa05203:Viral carcinogenesis	4	20	0.01777	PIK3CA, CASP3, CDK2, CDK1	20	205	6879
hsa04015:Rap1 signaling pathway	4	20	0.018943	FLT1, PIK3CA, MET, EGFR	20	210	6879
hsa05222:Small cell lung cancer	3	15	0.022516	PIK3CA, CDK2, PTGS2	20	85	6879
hsa04014:Ras signaling pathway	4	20	0.022985	FLT1, PIK3CA, MET, EGFR	20	226	6879
hsa04012:ErbB signaling pathway	3	15	0.023518	GSK3B, PIK3CA, EGFR	20	87	6879
hsa04066:HIF-1 signaling pathway	3	15	0.028248	FLT1, PIK3CA, EGFR	20	96	6879
hsa04114:Oocyte meiosis	3	15	0.036905	CCNB1, CDK2, CDK1	20	111	6879
hsa04726:Serotonergic synapse	3	15	0.036905	CASP3, PTGS2, PTGS1	20	111	6879
hsa04670:Leukocyte transendothelial migration	3	15	0.039367	PIK3CA, MMP2, MMP9	20	115	6879
hsa04919:Thyroid hormone signaling pathway	3	15	0.039367	GSK3B, PIK3CA, ESR1	20	115	6879
